# AI-assisted assessment of bowel preparation from patient-generated images for pre-procedure triage

**DOI:** 10.1038/s41598-026-49438-7

**Published:** 2026-04-24

**Authors:** Ye-Chan Kim, Hilal Hwang, Jong-Bub Lee, Seon-Min Lee, Si-Yeon Lee, Jin Wook Yi, Hyun-Gyu Lee

**Affiliations:** 1 Department of Electrical and Computer Engineering, Inha University, Incheon, Republic of Korea; 2 Department of Surgery, Inha University Hospital, Inha University College of Medicine, Incheon, Republic of Korea; 3 Department of Surgery, Yonsei University College of Medicine, Seoul, Republic of Korea; 4 Thyroid Cancer Center, AIN Hospital, Incheon, Republic of Korea; 5 College of Medicine, Inha University, Incheon, Republic of Korea

**Keywords:** Computational biology and bioinformatics, Gastroenterology, Health care, Medical research

## Abstract

Inadequate bowel preparation before colonoscopy or colorectal surgery can delay procedures, require repeat preparation, and increase clinical workload. Current assessment depends on manual review of patient-reported rectal effluent images by nursing staff, which is time-consuming and subject to inter-observer variation. To develop and explore the feasibility of an AI-assisted pre-procedure triage model that classifies bowel-preparation adequacy from patient-generated toilet images to support pre-colonoscopy assessment. A total of 1,508 patient-generated toilet images were retrospectively collected from colorectal surgery patients at a tertiary hospital (Aug 2019–Feb 2023). To avoid within-patient correlation, we retained one image per patient, excluding 490 images from patients with multiple submissions, resulting in 1,018 images from 1,018 unique patients. Three raters (resident, faculty surgeon, graduate student) independently labeled each image; disagreements were resolved by a two-of-three consensus rule. A DenseNet-201 model incorporating a Feature Pyramid Network (FPN) for multi-scale feature representation was trained to classify bowel preparation as *clean* or *not clean*. Model performance was assessed using stratified five-fold cross-validation on a development set (*n* = 778) and a held-out test set drawn from high-confidence, three-rater consensus cases (*n* = 240), using clinically relevant metrics including AUROC, F1-score, sensitivity, and specificity. The model achieved a mean AUROC of 0.886, F1-score of 0.882, and sensitivity of 0.931 for detecting inadequate preparation (10 repeated five-fold cross-validation; development set, *n* = 778). Grad-CAM visualization showed attention focused on residual stool and water turbidity, consistent with clinician interpretation. Ablation analysis indicated incremental improvements from random oversampling, smoothing weight decay, and FPN integration. The AI-assisted bowel-preparation triage model showed robust discrimination of inadequate preparation using real-world patient images. This approach has the potential to streamline manual review and enhance efficiency in pre-procedure assessment. Further studies should validate the model prospectively and evaluate its integration into clinical workflow.

**Supplementary Information:**

The online version contains supplementary material available at 10.1038/s41598-026-49438-7.

## Introduction

Inadequate bowel preparation remains a persistent operational challenge in gastrointestinal services, contributing to procedure delays, inefficient use of endoscopy rooms, and repeat examinations that increase healthcare costs and resource waste^1–4^. Despite standardized bowel-cleansing protocols, variability in patient adherence and physiological response continues to yield a significant proportion of inadequate preparations. This issue disrupts clinical throughput, reduces scheduling efficiency, and increases the workload burden on already constrained endoscopy units.

In routine practice, many hospitals rely on manual review of patient-generated toilet images submitted via telephone, messaging platforms, or hospital portals for pre-procedure assessment. However, this triage process depends heavily on subjective visual interpretation by nursing staff, making it difficult to maintain consistency across shifts and institutions. Prior work has demonstrated substantial inter-observer variability in evaluating bowel-preparation adequacy, even among physicians working in the same practice^1^. Furthermore, patient self-reports of bowel-preparation quality are unreliable, often overestimating adequacy and showing poor correlation with colonoscopic findings^5^. These limitations underscore the need for a reproducible and scalable assessment strategy to support pre-procedure decision-making.

Standardized scoring systems such as the Aronchick scale, the Ottawa Bowel Preparation Scale (OBPS), and the Boston Bowel Preparation Scale (BBPS) are widely used in clinical research and quality reporting^6,7^. Professional guidelines, including those of the U.S. Multi-Society Task Force on Colorectal Cancer, emphasize the importance of adequate cleansing to prevent avoidable repeat procedures^8^. However, these tools are applied during colonoscopy and offer no actionable feedback before the procedure. As a result, patients with inadequate preparation are often identified only after endoscope insertion, when re-preparation causes procedural delays and inefficient resource use.

Recent advances have demonstrated the potential of artificial intelligence (AI) to evaluate bowel cleanliness from colonoscopic images^9^. Smartphone-based interventions and text message-based AI systems have been developed to improve patient adherence to preparation instructions^10,11^. More recently, Zhu et al. proposed a deep learning system that classifies patient-submitted toilet images to assess bowel preparation adequacy^12^—an approach conceptually similar to the present work. Similarly, Lee et al. demonstrated that patient-captured stool images could be used with deep learning to predict endoscopic mucosal inflammation in ulcerative colitis^13^, further supporting the feasibility of patient-generated image classification in gastrointestinal care. However, none of these prior studies addressed pre-procedure triage workflow integration, inter-rater variability in ground-truth labeling, or model interpretability required for clinical deployment.

A practical clinical solution must not only provide acceptable predictive performance but also align with workflow efficiency, reduce repetitive manual review, and offer interpretable outputs that support clinical accountability. Rather than replacing clinical judgment, AI can function as a workflow-level decision-support tool for early identification of patients requiring re-dosing or additional hydration before colonoscopy. For such systems to be adopted in practice, transparency in model decision-making and reliable labeling strategies are essential.

In this study, we developed an AI-assisted pre-procedure triage model that classifies bowel-preparation adequacy using real-world, patient-generated toilet images. To improve labeling reliability and reflect pragmatic clinical decision-making, we applied a consensus annotation strategy using three raters with different levels of clinical expertise. We also incorporated model-interpretability analysis to enhance explainability and support clinical validation. The model was evaluated using clinically meaningful metrics to examine its feasibility for reducing manual review burden and supporting scalable triage within pre-colonoscopy workflows.

## Materials and methods

### Study design and ethical approval

**Fig. 1:**
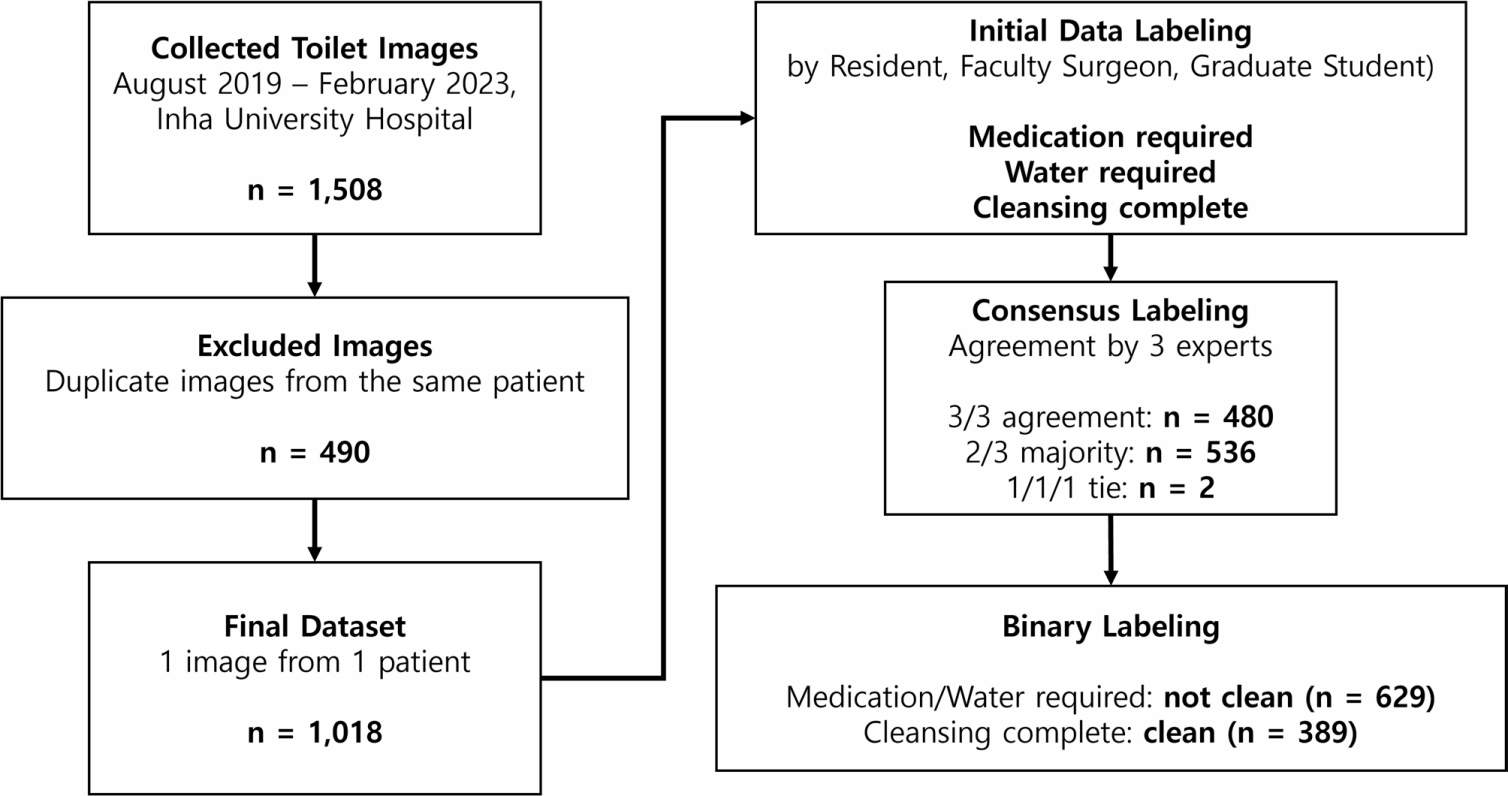
Data collection and labeling pipeline. After removing duplicate images per patient (*n* = 490) from 1,508 collected images, one image per patient was retained (*n* = 1,018). Three-rater consensus labeling (majority vote; resident retained for 1/1/1) produced three-class labels that were subsequently binarized into not clean (*n* = 629) and clean (*n* = 389).

This retrospective study was conducted at the Department of Colorectal and Anal Surgery, Inha University Hospital, between August 2019 and February 2023. This study was approved by the Institutional Review Board of Inha University Hospital, Incheon, Korea (IRB No. 2025-12-024) and was conducted according to the Declaration of Helsinki. The need for informed consent was waived by the Institutional Review Board of Inha University Hospital owing to the retrospective nature of the study. The overall data collection and labeling pipeline is summarized in Fig. 1.

### Dataset characteristics and image acquisition

A total of 1,508 toilet images were retrospectively collected from patients undergoing colorectal surgery (Aug 2019–Feb 2023). Because this was a retrospective study based on available clinical data, no formal sample size calculation was performed. To avoid within-patient correlation, we retained one image per patient; when multiple images were available for the same patient, we removed duplicates (*n* = 490, 32.5% of the original 1,508 images), resulting in a final dataset of 1,018 images from 1,018 unique patients.

All patient-submitted toilet images collected during the study period were eligible for inclusion; beyond duplicate removal, no additional exclusions were applied. Specifically, images were not excluded on the basis of poor image quality, non-interpretable content, or missing labels, as all submitted images were retained to preserve the real-world character of the dataset. Images were captured by patients using personal smartphones as part of routine pre-operative bowel preparation and submitted through standard clinical communication channels. The dataset reflects real-world variability in image quality, including differences in lighting conditions, camera devices, framing, and viewing angles commonly encountered in patient submissions.

Each image captured visual cues routinely used by clinical staff when assessing bowel-preparation adequacy, including effluent color, liquid turbidity, and visible residue. Images were initially categorized into three clinically actionable classes—medication required, water required, and cleansing complete—based on the need for further intervention before colonoscopy. For downstream analysis, images labeled as medication required or water required were grouped as not clean, while cleansing complete was labeled as clean, reflecting the primary pre-procedure decision point in clinical workflow.

**Fig. 2:**
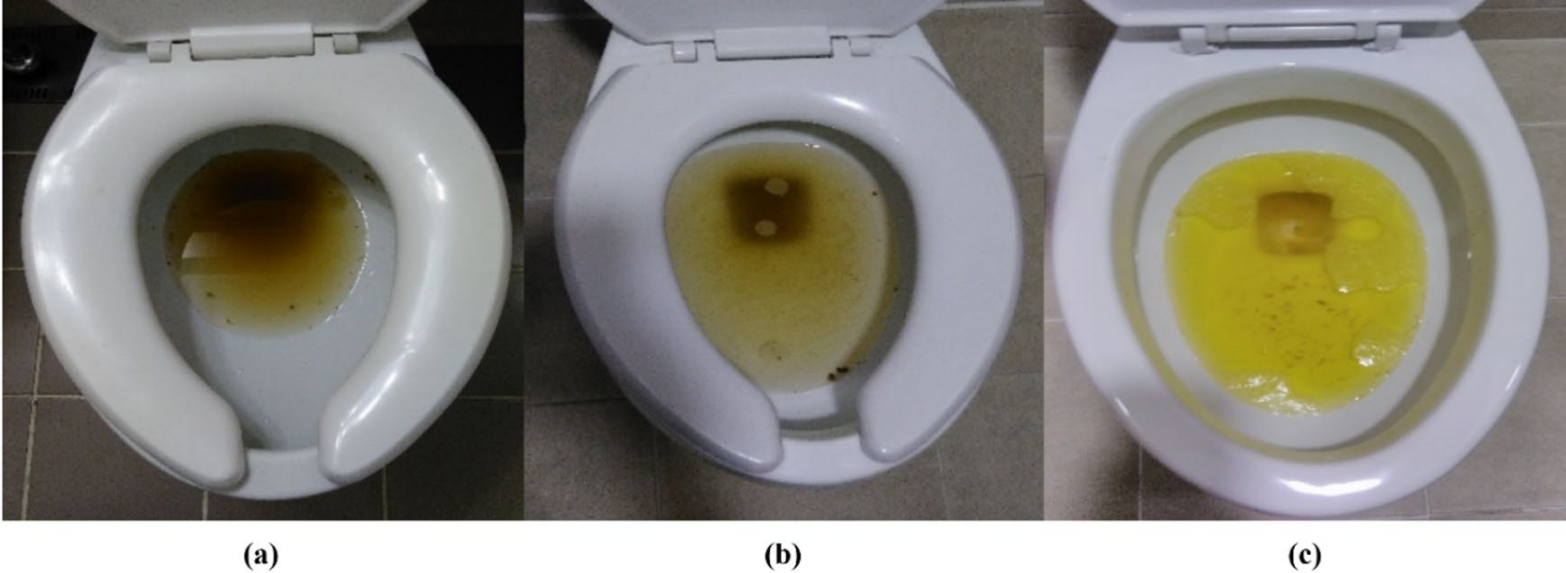
Representative images illustrating bowel-cleansing statuses. (**a**) Medication required; (**b**) Water required; (**c**) Cleansing complete.

The final dataset comprised 629 (61.8%) not-clean images and 389 (38.2%) clean images. Representative examples illustrating the diversity of bowel-cleansing status and image acquisition conditions are shown in Fig. 2.

### Labeling and consensus strategy

Labeling was performed independently by three raters with different levels of clinical training and perspectives: (1) a colorectal-surgery resident, (2) a faculty colorectal surgeon, and (3) a medical graduate student. All raters followed a predefined labeling guideline.

Each image was first evaluated using three visual sub-labels: effluent color, liquid turbidity, and visible residue. Effluent color was graded as brown or black (0), dark yellow (1), or yellow or clear (2). Turbidity was categorized as unclear or cloudy (0) versus clear or near-transparent (1). Residue was graded as large (0), moderate (1), or minimal or absent (2). All sub-labels were assigned independently, without access to other raters’ evaluations.

Based on these sub-labels, each image was assigned to an overall bowel-preparation class: Class 0 (additional medication required), Class 1 (additional water intake required), or Class 2 (no further intervention required). For model development and evaluation, this three-class scheme was further collapsed into a binary classification, with Classes 0 and 1 grouped as not clean and Class 2 as clean.

Disagreement among raters was resolved using a two-of-three majority rule. The resident’s label was retained only in cases with ambiguous image quality, following a predefined protocol. Among 1,018 images, 480 (47.2%) showed complete three-rater agreement and 536 (52.7%) were resolved by majority vote. Fully discordant cases (1/1/1) were rare (2 cases; 0.2%), and the resident-retained rule was therefore invoked only in these instances (Supplementary Table S1). Agreement with the consensus labels was 0.888 for the resident, 0.754 for the faculty surgeon, and 0.987 for the graduate student (see Supplementary Figure S1 for detailed comparisons). The consensus labels were used as the reference standard for model development and evaluation.

Inter-rater reliability among the three raters was quantified using Fleiss’ kappa. We computed kappa for the original three-class labels (0 = medication required, 1 = water required, 2 = cleansing complete) and for the derived binary labels (not clean vs. clean). 95% confidence intervals were estimated by bootstrap resampling (1,000 replicates). Fleiss’ κ was 0.443 (95% CI 0.406–0.475) for the three-class labels and 0.446 (95% CI 0.405–0.484) for the binary labels.

**Fig. 3:**
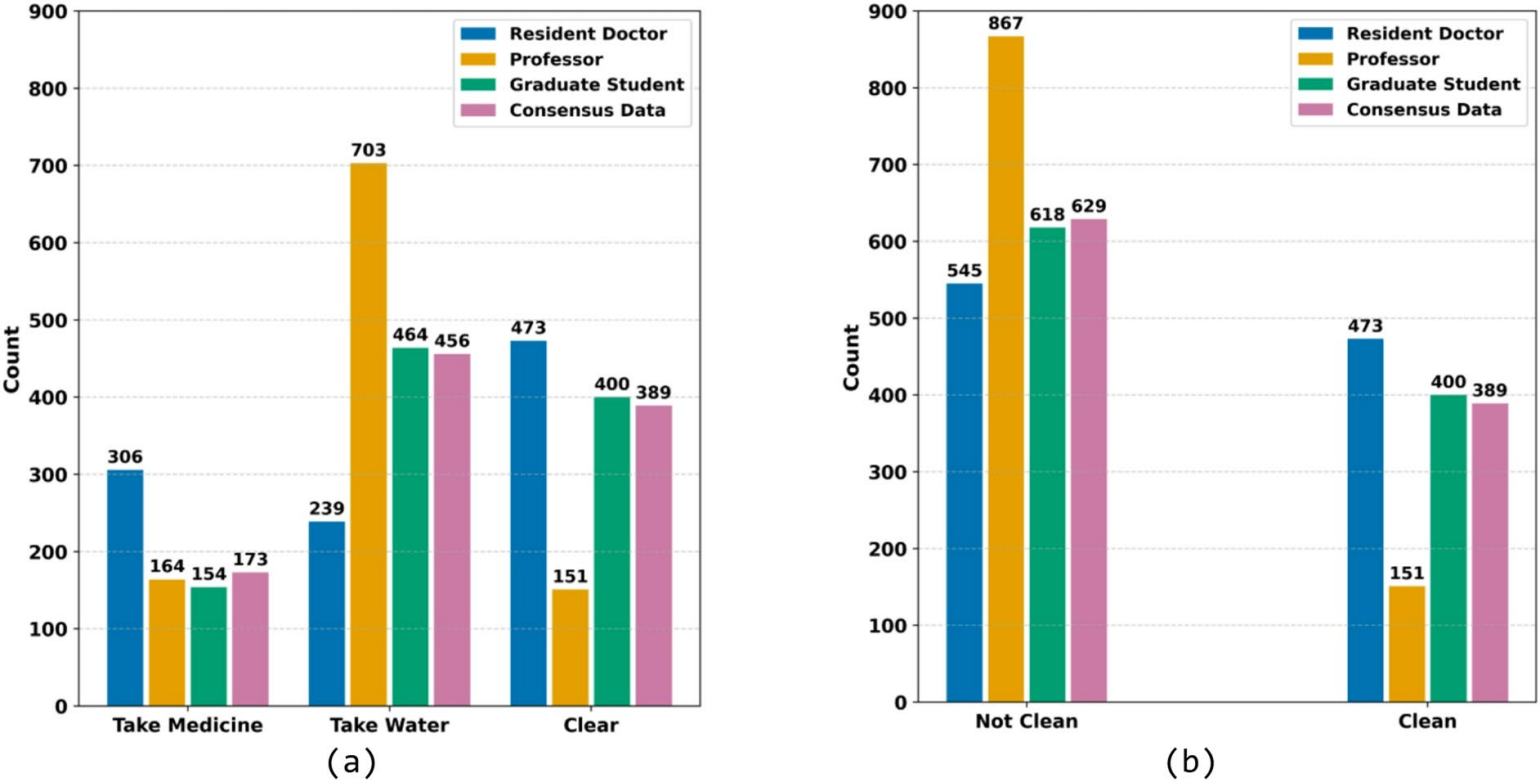
Label distribution of the bowel-preparation dataset. Shown are the label distributions of 1,018 patient-captured images as annotated by three medical experts and the consensus reference. Colors represent raters: blue, resident physician; orange, professor; green, graduate student; pink, consensus data. (**a**) Distribution under the original three-class labeling (*medication required*, *water required*, *cleansing complete*) and (**b**) distribution under the binary-class labeling (*not clean* / *clean*).

To assess robustness to labeling uncertainty, we conducted a sensitivity analysis under three adjudication scenarios: (S0) the primary consensus strategy applied to all 1,018 images; (S1) strict majority vote with fully discordant cases excluded (*n* = 1,016); and (S2) training restricted to images with complete three-rater agreement (*n* = 480), with the held-out test set identical across all scenarios. As fully discordant cases represented only 2 of 1,018 images (0.2%), a faculty-weighted adjudication scenario was not separately evaluated. Accordingly, the resident-retained rule had negligible impact on class distribution and model performance, as confirmed by the sensitivity analysis in Supplementary Table S2. Label distributions across all raters and the consensus reference are shown in Fig. 3.

### Model architecture and training procedure

**Fig. 4:**
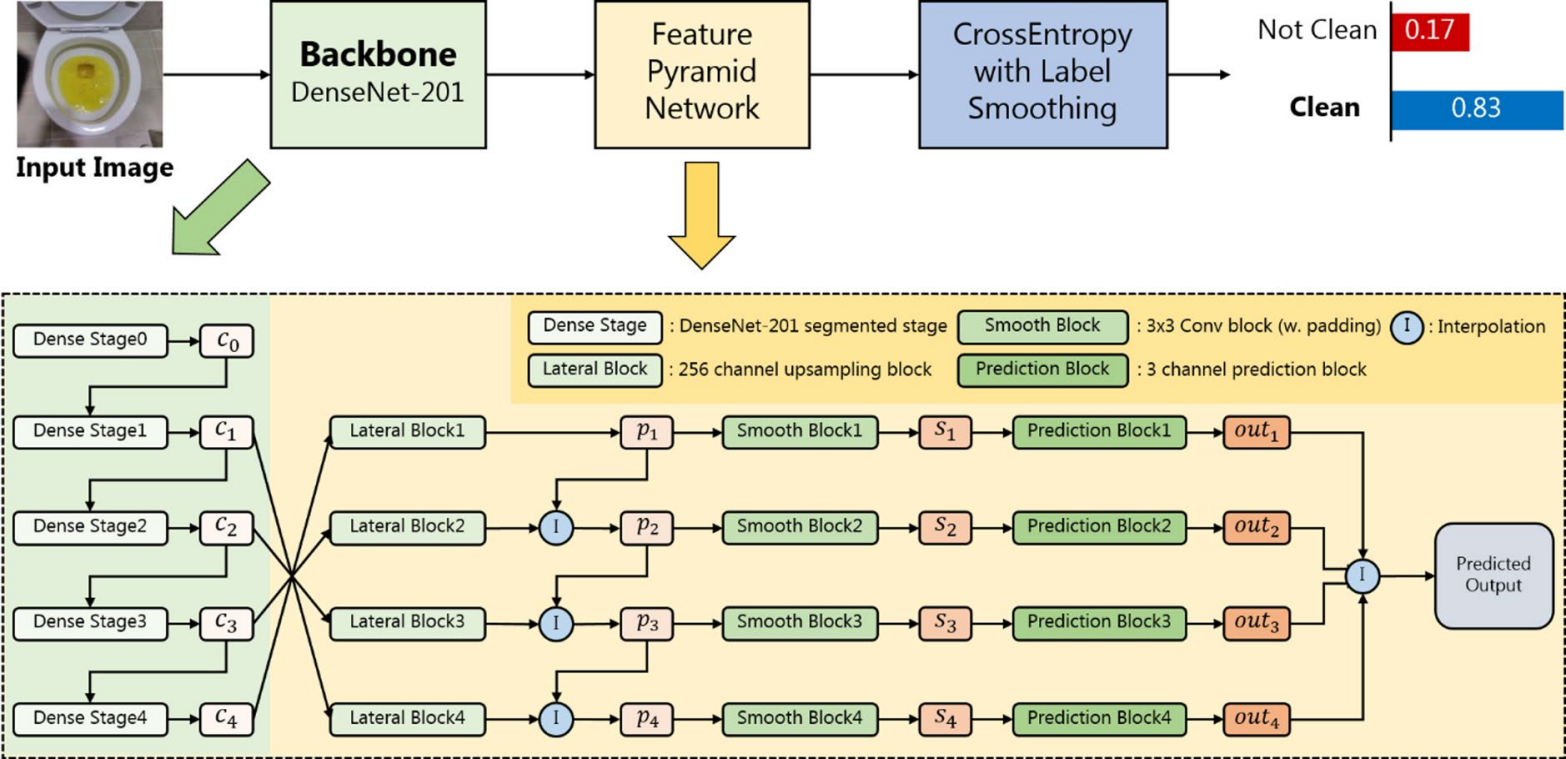
Architecture of the proposed deep-learning model. The model consists of a DenseNet-201 backbone connected to a Feature Pyramid Network (FPN) for multi-scale feature extraction. Each lateral block produces intermediate feature maps that are upsampled and aggregated to form the final prediction. Label smoothing is applied during training to stabilize optimization and reduce overfitting. The final output provides the probability of each class (*not clean* / *clean*), which is used for binary classification of bowel-preparation images.

The classification model used a DenseNet-201 backbone combined with a Feature Pyramid Network (FPN) for multi-scale feature extraction, as shown in Fig. 4. DenseNet-201 was selected as the backbone because densely connected architectures promote feature reuse and have shown strong performance in medical image classification^14^. An FPN was added to capture visual patterns at multiple spatial scales^15^, which was considered advantageous for bowel-preparation images with heterogeneous turbidity and residue patterns. In preliminary architecture comparisons, DenseNet-201 with FPN showed the best overall performance (Supplementary Table S3).

Input images were resized to 512 × 512 pixels and converted from BGR to RGB color space. Data augmentation consisted of horizontal flipping (*p* = 0.3) and vertical flipping (*p* = 0.3). No additional normalization was applied. Although standard ImageNet normalization is commonly used with pretrained models, it was omitted here because the DenseNet-201 backbone was fine-tuned end-to-end on the target dataset, allowing the network to adapt its internal representations to the input distribution during training. Training employed the Adam optimizer with the default PyTorch settings except for the learning rate (0.0001) and weight decay (0.0001)^16^, using a batch size of 16 for 50 epochs with label smoothing (ε = 0.1)^17,18^. No learning rate scheduling, early stopping, or dropout was used. Random oversampling was applied to balance classes^19^.

Implementation used Python 3.8 and PyTorch on an NVIDIA RTX 3090 GPU. The model outputs probabilities between “clean” and “not clean,” serving as a decision-support signal for nursing triage and not intended for autonomous diagnostic use.

### Model evaluation protocol

Model performance was assessed using stratified five-fold cross-validation on a development set. After consensus labeling, we first identified a high-confidence pool consisting of images with complete three-rater agreement on the original three-class label (3/3 agreement; *n* = 480; not clean: 351, clean: 129). From this pool, we selected a held-out test set of 240 images (not clean: 175, clean: 65) using stratified random sampling by the binary label (seed = 1004). The remaining 778 images (not clean: 454, clean: 324) comprised the development set. Within the development set, we performed stratified 5-fold cross-validation based on the binary label (seed = 1004) to preserve class proportions across folds. Because exactly one image per patient was retained, the number of images equals the number of patients in all splits and folds. We verified that no patient identifiers overlapped between training and validation folds, and that the held-out test set contained no overlapping patient identifiers with the development set.

Evaluation metrics included accuracy, F1 score, sensitivity, specificity, and AUROC, and performance was summarized as the mean ± standard deviation across 10 repeated five-fold cross-validation experiments (50 runs in total). The numbers of samples and class distributions for the training, validation, and held-out test splits are summarized in Supplementary Table S4. Model calibration was assessed on the held-out test set using the Brier score, calibration plot^20^, and logistic calibration regression, in which predicted probabilities were compared against observed outcome frequencies across ten equal-width bins. Calibration slope and intercept were estimated by fitting a logistic regression model to the logit-transformed predicted probabilities against observed binary outcomes, with slope = 1.0 and intercept = 0.0 indicating perfect calibration. As PPV and NPV vary with the prevalence of inadequate preparation, estimated values across a range of hypothetical prevalence scenarios are reported in Supplementary Table S5.

As the held-out test set was drawn exclusively from images with complete three-rater agreement, it represents a high-confidence benchmark that minimises label noise; however, it may over-represent visually unambiguous cases. No model ensembling or retraining on the full development set was performed.

### Explainability and visualization method

Model interpretability was assessed using Gradient-weighted Class Activation Mapping (Grad-CAM)^21^ to visualize spatial regions contributing to model predictions. Grad-CAM was applied to the final convolutional layer of DenseBlock4 for the baseline DenseNet-201, and to the FPN output layer for the FPN-augmented model. This analysis was used to examine whether model attention aligned with clinically relevant visual cues — namely, residual stool and water turbidity — and to assess whether multi-scale feature integration influenced the spatial focus of model predictions.

### Statistical and reproducibility considerations

All experiments were conducted with fixed random seeds to ensure reproducibility. Statistical analyses used Python (Numpy, Scipy, scikit-learn). The source code for model training, evaluation, and inference is publicly available at GitHub. Anonymized sample images may be made available to qualified researchers upon reasonable request to the corresponding author, subject to institutional review board approval and the execution of a data use agreement.

### Ethical and privacy compliance

All images were captured by patients using personal smartphones and stored in a secure, hospital-local repository. No personally identifiable information or metadata (e.g., timestamps, device IDs, or record numbers) were collected; only the anonymized images and their corresponding labels were used for analysis. Data handling complied with the Personal Information Protection Act of South Korea and HIPAA-equivalent privacy standards^22^. The AI model was developed as a proof-of-concept decision-support tool, not intended for autonomous diagnostic use.

## Results

### Model performance

The proposed DenseNet-201 + FPN configuration achieved robust classification accuracy across five-fold cross-validation (Table 1). The model yielded a mean AUROC of 0.886 ± 0.031, F1-score of 0.882 ± 0.026, sensitivity of 0.931 ± 0.031, specificity of 0.840 ± 0.072, and overall accuracy of 0.906 ± 0.020, all derived from 10 repeated five-fold cross-validation on the development set (*n* = 778; 50 runs total). The AUROC indicates strong discrimination between adequate and inadequate preparations, while the F1-score reflects balanced precision and recall despite class imbalance.

Unless otherwise stated, all primary performance metrics reported in this section were computed at a default classification threshold of 0.50; threshold sensitivity analysis across alternative cutpoints is presented separately in the Discussion. The detailed breakdown of classification outcomes on the held-out test set from a single-run evaluation using the Fold 0 model is provided in Supplementary Table S6. To avoid ambiguity across reported results, the main Results (Table 1) and Supplementary Table S7 present the primary model performance based on 10 repeated five-fold cross-validation (50 runs), whereas Supplementary Table S3 reports single-run results used for architecture selection, and Supplementary Table S2 summarizes sensitivity analyses under alternative adjudication strategies using a single five-fold run. Accordingly, Table 1 should be considered the primary reference for model performance, while supplementary tables provide supporting analyses under specific experimental conditions.

Compared with the baseline DenseNet-201, FPN integration resulted in modest gains (AUROC + 0.006, F1 + 0.021), suggesting that multi-scale feature aggregation may improve detection of subtle turbidity and residual-stool patterns. Detailed architecture and ablation results are summarized in Supplementary Tables S3 and S7. The differences in specificity and accuracy between Supplementary Table S3 and Table 1 and Supplementary Table S7 arise from the use of a single five-fold cross-validation run in Table S3 for architecture comparison, whereas Table 1 and Table S7 report results averaged over 10 repeated five-fold cross-validation runs, providing more stable performance estimates.

Sensitivity analysis across adjudication scenarios demonstrated stable performance on the held-out test set. Excluding fully discordant cases (S1) produced negligible differences from the base model (AUC: 0.885 vs. 0.887; F1: 0.878 vs. 0.880). Training on high-confidence labels only (S2) yielded comparable overall performance (AUC: 0.879) with higher sensitivity (0.955) and lower specificity (0.803), a shift attributable in part to the reduced training set size (*n* = 240) rather than label quality alone. These findings indicate that model performance is robust to variation in adjudication strategy.

Model calibration on the held-out test set was assessed using multiple metrics. The Brier score was 0.077 (Brier skill score: 0.608; uninformative baseline: 0.196), indicating substantially better calibration than an uninformative baseline. The calibration intercept was − 0.099, indicating minimal overall probability bias (calibration-in-the-large). The calibration slope was 1.566, suggesting moderate overconfidence whereby predicted probabilities were more extreme than observed outcome frequencies. The calibration plot (Supplementary Figure S2) demonstrated general alignment between predicted probabilities and observed outcome frequencies, with minor deviations in the low-probability region attributable to sparse bin occupancy. This model is designed as a threshold-based binary classifier that outputs a fixed clean/not-clean decision rather than a calibrated probability estimate for clinical use. Accordingly, the observed overconfidence does not affect the intended triage output. However, it is relevant to any deployment scenario in which the raw probability output — rather than the binary classification — is used to inform graded clinical decisions; this point is discussed further in the Limitations section.

As PPV and NPV vary with the prevalence of inadequate preparation, estimated values across hypothetical scenarios are reported in Supplementary Table S5. NPV remained high across all scenarios (0.824–0.990), indicating that images classified as clean are unlikely to represent inadequate preparation. PPV was more sensitive to prevalence, ranging from 0.409 at 10% prevalence to 0.936 at 70%. At the observed prevalence in this dataset (61.8%), estimated PPV and NPV were 0.910 and 0.871, respectively.

**Table 1:** Comparison of binary consensus data performance according to deep learning models. All metrics represent mean ± SD from 10 repeated five-fold cross-validation experiments (50 runs total) on the development set (*n* = 778). The backbone models were selected based on their performance on the development set. Single-run held-out test set results (*n* = 240) are reported separately in Supplementary Table S6.

Metric	DenseNet-201 + FPN (final model)	Improvement vs. baseline DenseNet-201 (Δ)	Clinical implication
AUROC	0.886 ± 0.031	+ 0.006	Reliable discrimination of inadequate prep before procedure
F1 score	0.882 ± 0.026	+ 0.021	Balanced precision / recall for triage consistency
Sensitivity	0.931 ± 0.031	+ 0.015	Early detection of insufficient cleansing
Specificity	0.840 ± 0.072	-0.005	Low false-positive rate reduces unnecessary re-dosing
Accuracy	0.906 ± 0.020	+ 0.009	Overall workflow-level reliability

### Explainability and visualization

**Fig. 5:**
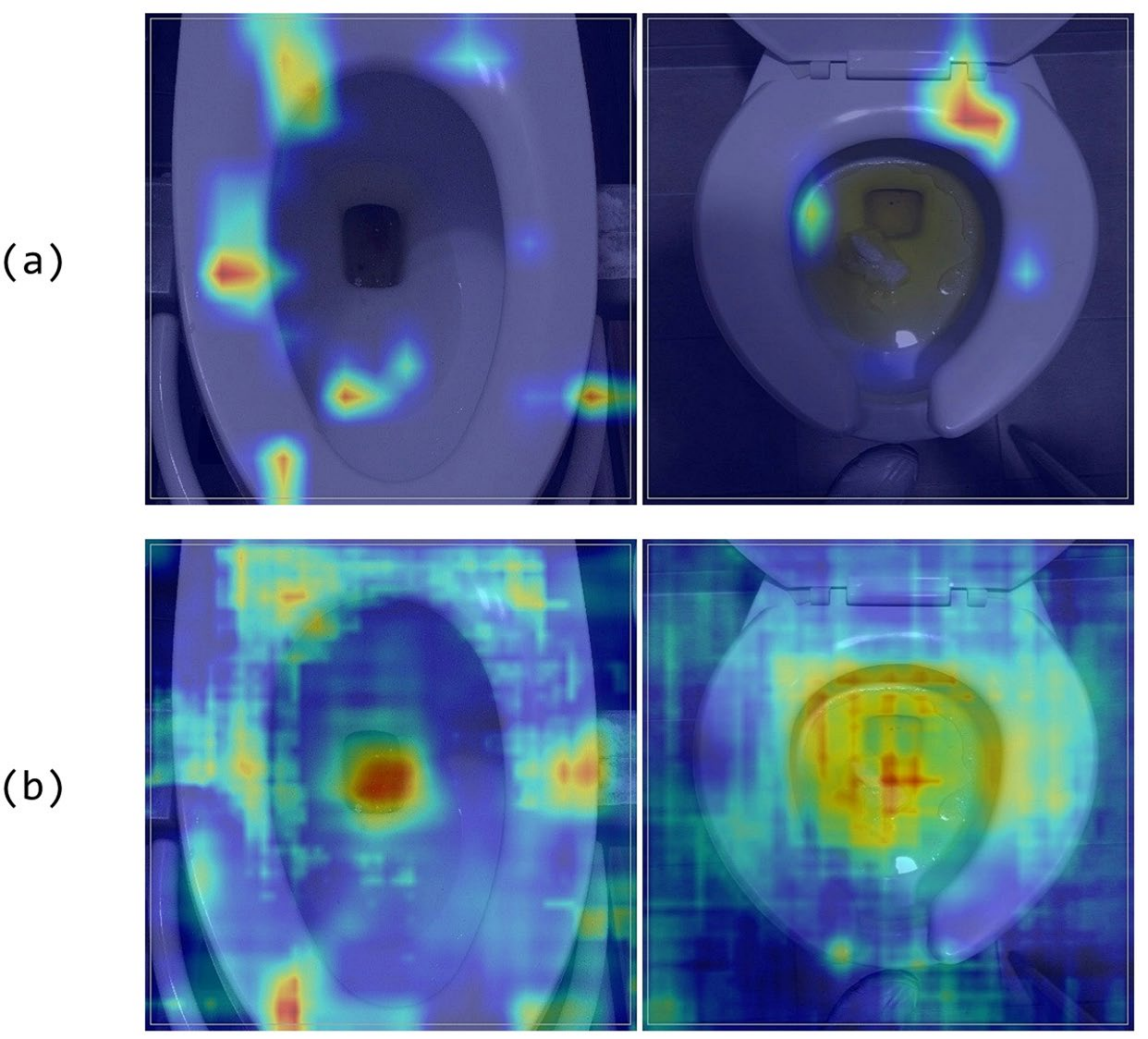
Comparison of Grad-CAM visualizations from DenseNet-201 block4 and the FPN. (**a**) The Grad-CAM from the last DenseBlock4 showed partial attention to the bowl interior but also emphasized peripheral regions such as the toilet rim. (**b**) The FPN-based Grad-CAM exhibited more concentrated attention on residual stool and turbid water within the bowl, features that are clinically relevant for evaluating cleansing adequacy.

Grad-CAM heatmaps (Fig. 5) demonstrated that both the baseline DenseNet-201 and the FPN-augmented model primarily attended to bowel contents within the toilet bowl, highlighting residual stool and water turbidity—features consistent with clinician visual reasoning. The addition of the Feature Pyramid Network (FPN) modestly refined this attention pattern by reducing peripheral emphasis on background areas such as the toilet rim and by enhancing focus on the interior region relevant to cleansing adequacy. Although the overall predictive improvement was small, this shift in spatial attention supports the interpretability of the FPN integration and provides visual transparency regarding the model’s decision process. Additional representative Grad-CAM examples illustrating successful and failed localizations are provided in Supplementary Figure S3.

### Error cases analysis

Error analysis revealed two primary failure modes observed across misclassified cases. On the held-out test set, the model produced 9 false positive and 12 false negative cases out of 240 images (Supplementary Table S6). False positive errors were characterised by images containing interfering visual elements — such as foam, tissue paper, or heavily discoloured water — that mimicked the appearance of inadequate preparation despite being adjudicated as adequate. The model appeared to overweight these distracting cues, leading to incorrect not-clean predictions in visually complex but clinically acceptable cases.

False negative errors showed a complementary pattern, in which the model failed to detect water turbidity or residual soiling present in the images, incorrectly predicting adequate preparation. This failure mode carries greater clinical significance, as it may result in patients proceeding to colonoscopy without sufficient bowel cleansing. In many of these cases, turbidity was subtle or exhibited low contrast against the background, suggesting that the model was insufficiently sensitive to these cues under certain image conditions. Representative cases of each error type are illustrated in Supplementary Figure S4.

Both failure modes likely reflect the inherent labelling ambiguity in borderline cases, as evidenced by the moderate inter-rater agreement in this study (Fleiss’ κ = 0.443–0.446). Misclassified cases predominantly involved low-confidence predictions, consistent with the observation that errors tend to cluster near the decision boundary.

## Discussion

This study demonstrates the feasibility of an AI-assisted pre-procedure triage system that automatically evaluates bowel-preparation adequacy from patient-captured toilet images. By integrating a DenseNet-201 backbone with a Feature Pyramid Network, the proposed model achieved stable discrimination under heterogeneous real-world imaging conditions. Unlike prior AI approaches that focus on endoscopic or intra-procedural images, this work targets the pre-colonoscopy phase, where early identification of inadequate preparation has the potential to reduce downstream delays and avoid unnecessary repeat procedures.

### Clinical and workflow implications

The proposed system addresses a persistent operational bottleneck in gastrointestinal services, where manual review of patient-generated images is labor-intensive, variable across nursing shifts, and prone to subjective inconsistency. The AI-assisted triage framework positions automated image classification upstream of colonoscopy scheduling, allowing nursing staff to focus attention on cases flagged as not clean while routine cases proceed without additional review.

In this configuration, AI outputs function as pre-screening signals rather than definitive decisions. Clinician oversight is preserved through selective review of flagged cases and confirmation before final EMR entry. By narrowing the scope of manual review, this workflow has the potential to reduce redundant effort and improve decision consistency without altering established clinical accountability structures. Although this study did not prospectively quantify time savings or workload reduction, the conceptual integration demonstrates a feasible pathway for embedding AI-assisted triage into existing pre-procedure processes.

Importantly, the system is designed to support nurse-led triage. By operating upstream and providing structured, image-based signals, the model aligns with human-in-the-loop principles that are critical for safe deployment in routine clinical settings.

As a proof-of-concept system, the model is not yet ready for autonomous clinical deployment and should be considered a decision-support aid rather than a replacement for clinical judgment. Nevertheless, the discriminative performance demonstrated here is promising and justifies further investigation through prospective evaluation and external validation in diverse clinical settings. At the default threshold of 0.50, the model trained on Fold 0 achieved a sensitivity of 0.931 and NPV of 0.824 on the held-out test set (full confusion matrix in Supplementary Table S6). The five-fold mean on the same test set was 0.922 ± 0.010 for sensitivity and 0.852 ± 0.044 for specificity (Supplementary Table S2), with the inter-fold variability reflecting differences in learned weights rather than different test data. The wider SD for specificity relative to sensitivity is consistent with the small size of the clean class in the test set (*n* = 65), which makes specificity disproportionately sensitive to fold-level differences in the learned decision boundary; this is discussed in detail in the Supplementary Table S6 footnote.

The default threshold of 0.50 was selected because false negatives — patients with inadequate preparation incorrectly cleared as ready — carry greater clinical risk than false positives, as proceeding to colonoscopy without sufficient bowel cleansing may result in missed lesions, procedural interruption, or the need for repeat examination. Threshold analysis across a range of cutpoints (Supplementary Figure S5) demonstrated that in settings where minimizing false negatives is the primary clinical priority, lowering the threshold to 0.30 yields sensitivity of 0.954 while maintaining specificity of 0.815 — the highest specificity achievable at sensitivity ≥ 0.95 in this dataset. Threshold selection should therefore be guided by the clinical risk tolerance of the deployment setting rather than fixed a priori. Table 2 summarizes performance at three clinically relevant thresholds on the held-out test set (single-run evaluation, *n* = 240).

**Table 2:** Performance of the DenseNet-201 + FPN model at three clinically relevant classification thresholds (held-out test set, Fold 0 model; single-run evaluation, *n* = 240).

Threshold	Sensitivity	Specificity	NPV
0.30	0.954	0.815	0.869
0.50	0.931	0.862	0.824
0.55	0.926	0.892	0.817

Should the model progress toward clinical integration, post-deployment performance monitoring would be necessary to detect distributional shift arising from changes in patient population, preparation protocols, or image acquisition practices. Furthermore, a governance framework that includes periodic performance auditing, criteria for model retraining or updating, and clear protocols for escalation when model outputs are uncertain or inconsistent would be essential components of responsible clinical deployment. These considerations highlight the importance of a cautious, stepwise approach to clinical translation, in which AI outputs serve as one input among several in the triage decision-making process.

Evaluation of real-world workflow impact, including quantification of nurse review time, repeat preparation rates, and procedure cancellation rates, would require a prospectively designed implementation study and lies beyond the scope of this retrospective technical validation.

### Interpretability and annotation variability

The relatively high disagreement among the three raters reflects the inherent ambiguity of patient-generated toilet images rather than annotation error alone. Variations in lighting, camera angle, framing, and image quality frequently obscure key visual cues such as residual stool and water turbidity, making strict visual categorization challenging even for experienced clinicians. In this context, annotation variability arises not only from image conditions but also from differences in how raters operationalize visual criteria during decision-making.

Raters with extensive clinical experience may apply holistic or experience-driven heuristics, integrating contextual impressions beyond explicitly defined visual thresholds. In contrast, annotations that adhere closely to predefined labeling guidelines and sub-label criteria tend to show reduced variability in visually ambiguous cases. This difference in annotation strategy likely explains why the graduate student’s labels showed the highest agreement with the consensus dataset. Importantly, this observation does not imply superiority of one rater group over another, but rather highlights how different cognitive strategies interact with ambiguous visual inputs. Nevertheless, a potential limitation warrants explicit acknowledgment: the graduate student was the minority (odd-rater) in only 5.4% of majority-vote cases, while achieving a consensus agreement of 0.987. This pattern suggests that the majority-vote consensus may effectively approximate the graduate student’s individual labeling decisions rather than representing a balanced integration of all three raters’ perspectives. This should be considered when interpreting the consensus as a reference standard, and future studies should employ a larger and more diverse rater panel to mitigate this risk.

Given this inherent annotation variability, interpretability analysis was used to examine whether the model relied on stable visual cues shared across raters rather than rater-specific heuristics. Grad-CAM visualizations confirmed that model attention aligned with the core visual criteria defined in the annotation guidelines, as described in Sect.  3.2. This alignment suggests that model predictions are driven by consistent, annotation-relevant features rather than spurious background artifacts.

Under challenging conditions such as low illumination or partial occlusion, occasional mislocalized activations were observed, underscoring the limits of visual information in patient-generated images. These observations indicate that explainability analysis can serve as a practical auditing mechanism to identify failure-prone scenarios and inform refinements in image acquisition guidance or preprocessing, particularly in real-world deployment.

### Comparative perspective

Previous studies have applied AI to assess bowel cleanliness during colonoscopy or employed mobile interventions to improve preparation compliance. The present work extends this literature by demonstrating that patient-captured images—despite their uncontrolled acquisition conditions—can support reliable classification when combined with structured annotation and multi-scale feature representation. By focusing on pre-procedure triage rather than intra-procedural assessment, this approach addresses a distinct and underexplored stage in the clinical workflow. More broadly, the findings align with the growing use of patient-generated health data (PGHD) in outpatient and telehealth settings, suggesting feasibility beyond single-center deployment.

### Limitations and future directions

This study has several limitations. First, it was conducted as a single-center retrospective analysis, which may limit generalizability to settings with different patient populations, preparation agents, or clinical workflows. Nevertheless, several features of the present dataset support a degree of clinical realism. The dataset spans four years (2019–2023), encompassing temporal variation in smartphone devices, patient submission habits, and preparation practices across multiple clinical cohorts. Furthermore, patient-generated toilet images are inherently less dependent on institution-specific endoscopic equipment or procedural protocols than intra-procedural images, such that inter-institutional variability is expected to be driven primarily by differences in imaging environment (e.g., lighting, device) rather than clinical setting per se. Finally, all images were collected under routine clinical care rather than a controlled research protocol, preserving the unselected, real-world character of the data. Despite these considerations, temporal holdout validation and multi-institutional external validation remain necessary to establish generalizability and be addressed in future research.

Second, patient-level clinical metadata such as demographics or preparation regimens were not available, precluding subgroup analyses by age, sex, or preparation type. Such analyses are needed to determine whether performance is consistent across patient subgroups. Similarly, image acquisition factors such as lighting conditions, camera device, framing, and stool consistency were not systematically recorded or quantified, precluding formal analysis of their impact on model performance. Prospective data collection with structured metadata on image acquisition conditions would be necessary to assess and mitigate these sources of variability.

Third, the held-out test set was sampled from a high-confidence pool with complete three-rater agreement, which may over-represent visually unambiguous cases. Test performance should therefore be interpreted as an optimistic upper-bound benchmark, as the test set was drawn exclusively from the most visually unambiguous cases; performance on consecutively collected, real-world submissions is likely to be lower, and prospective evaluation is needed to estimate true deployment performance.

Fourth, the calibration slope of 1.566 indicates moderate overconfidence in predicted probabilities, whereby the model’s probability outputs are more extreme than the observed outcome frequencies. In a clinical triage context where downstream decisions—such as re-dosing, procedure delay, or patient reassurance—may depend on the predicted probability rather than the binary classification alone, systematic overconfidence is clinically relevant. Post-hoc calibration methods such as Platt scaling, temperature scaling, or isotonic regression were not applied in this study. As this model is designed as a threshold-based binary classifier — outputting a fixed clean/not-clean decision rather than a calibrated probability for clinical use — the primary evaluation focused on discriminative performance rather than probability-based decision-making. Applying and validating such recalibration techniques should be addressed before any deployment in settings where calibrated probability estimates inform clinical actions.

As this study was designed as a single-center proof-of-concept technical validation, a prospective evaluation of real-world clinical impact was beyond its intended scope. Future work should include prospective, multi-institutional validation to assess real-world impact on workflow efficiency and patient outcomes, as well as quantitative evaluation of explainability as part of deployment monitoring. Integration with secure clinical systems, along with continued attention to privacy protection and regulatory alignment, will be essential to ensure ethical and transparent implementation. With continued refinement, AI-assisted triage models of this kind may function as workflow-embedded decision-support tools that facilitate proactive and data-driven preparation management before colonoscopy.

## Conclusion

This study developed and evaluated a deep learning model for AI-assisted pre-procedure triage that classifies bowel-preparation adequacy from patient-captured toilet images. The model integrates a DenseNet-201 backbone with a Feature Pyramid Network and demonstrated stable performance across heterogeneous real-world imaging conditions. This work demonstrates how automated image-based triage can reduce redundant manual review and support consistent, timely decision-making in pre-colonoscopy management.

Grad-CAM analysis indicated that the model relied on clinically relevant visual cues, providing transparent insight into model behavior and supporting interpretability in the context of pre-procedure assessment. This approach exemplifies how patient-generated health data (PGHD) can be transformed into actionable, privacy-compliant information that complements existing clinical workflows rather than replacing clinical expertise. Future studies should address multi-center prospective validation, integration of clinical metadata and temporal trends, and evaluation of usability and regulatory readiness to ensure reliable and transparent implementation within digital health infrastructure.

## Supplementary Information

Below is the link to the electronic supplementary material.

Supplementary Material 1

## Data Availability

The dataset used in this study consists of patient-generated toilet images collected under routine clinical care and cannot be publicly released due to patient privacy restrictions and institutional data governance policy. De-identified data may be made available to qualified researchers upon reasonable request to the corresponding author, subject to institutional review board approval and the execution of a data use agreement.

## Code availability

The source code for model training, evaluation, and inference is publicly available at https://github.com/Zrot77/bowel-prep-ai. The repository includes training scripts, evaluation pipelines, and inference code with documentation to support reproducibility.
