# Supplementary material for: AI-assisted assessment of bowel preparation from patient-generated images for pre-procedure triage
Source: Sci Rep. 2026 Apr 24;16:19099. doi: 10.1038/s41598-026-49438-7 (PMC13280243; doi:10.1038/s41598-026-49438-7)
Supplement: Supplementary file 1 — Supplementary Material 1 [file 41598_2026_49438_MOESM1_ESM.docx]

**Supplementary Table S1**. Three-rater agreement summary on the original 3-class labels

**Panel A**. Agreement strata across all images (N = 1,018)

| **Agreement stratum** | **Definition** | **n** | **% of total** |
| --- | --- | --- | --- |
| 3/3 agreement | All three raters assigned the same 3-class label | 480 | 47.2% |
| 2/3 majority agreement | Two raters agreed; majority label used | 536 | 52.7% |
| 1/1/1 disagreement | All three raters assigned different labels | 2 | 0.2% |
| **Total** |  | **1,018** | **100%** |

**Panel B.** Odd-rater distribution among 2/3 majority cases (N = 536)

| **Odd rater (minority label)** | **n** | **% of 2/3 cases** |
| --- | --- | --- |
| Faculty colorectal surgeon | 261 | 48.7% |
| Colorectal-surgery resident | 246 | 45.9% |
| Medical graduate student | 29 | 5.4% |
| **Total** | **536** | **100%** |

Original 3-class labels were defined as **0 = medication required, 1 = water required,** and **2 = cleansing complete**. Fully discordant cases (1/1/1; n = 2) were resolved using the prespecified resident-retained rule. Percentages may not sum to 100% due to rounding.

**Supplementary Figure S1.** Label agreement between individual medical experts and the consensus reference.


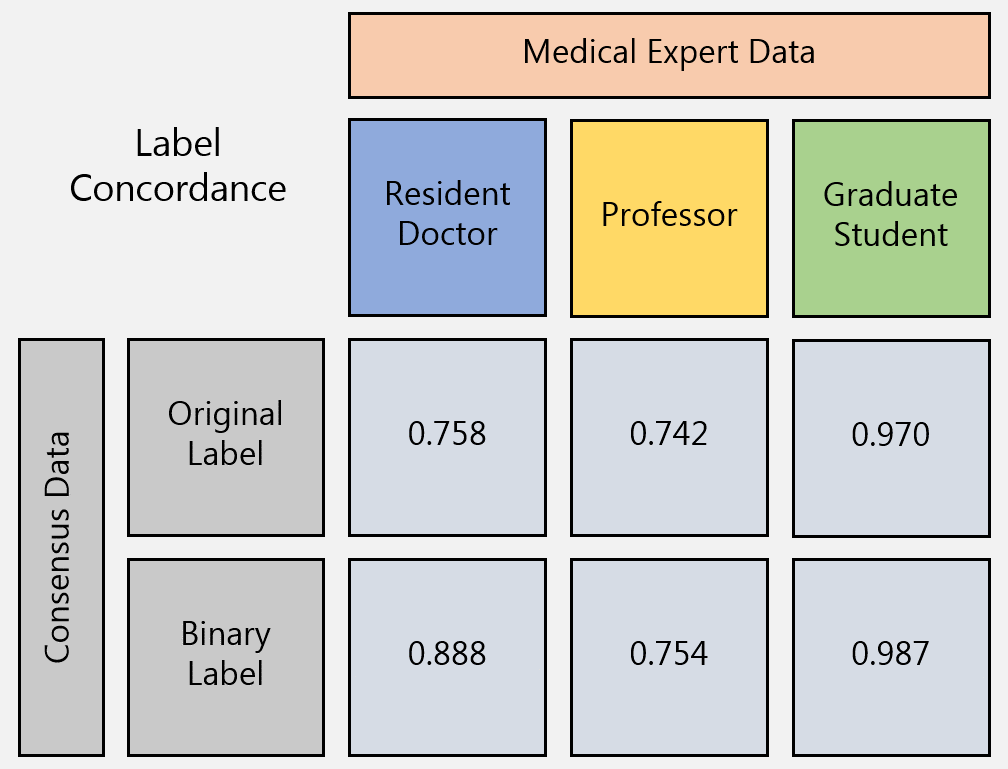


The figure shows the degree of label agreement between three medical experts and the consensus dataset for bowel-cleansing status.
“Original Label” represents the three-class categorization (*medication required*, *water required*, *cleansing complete*), and “Binary Label” represents the two-class version (*clean* / *not clean*).

Resident physicians applied more flexible thresholds for cleanliness, classifying marginal cases as *clean* and achieving a binary-label agreement of 0.888 with the consensus. Professors adopted stricter criteria, labeling more cases as *not clean* and showing the lowest agreement (0.754). Graduate students demonstrated the highest agreement (0.987), which indicates that the consensus threshold—formed by combining the three raters’ judgments—was closest to their decision boundary.

These findings highlight that different professional perspectives yield variable yet complementary definitions of bowel cleanliness. The consensus dataset functions as a practical reference standard rather than a fixed gold standard. However, as discussed in §4.2, the high agreement between the graduate student's labels and the consensus (0.987), combined with the graduate student's low odd-rater rate (5.4%), suggests that the consensus may disproportionately reflect one rater's labeling strategy. This limitation should be considered when interpreting the consensus labels.

**Supplementary Table S2.** Adjudication Sensitivity Analysis

| **Scenario** | **Labeling Strategy** | **N (train/val)** | **Test Acc** | **Test F1** | **Test Sens** | **Test Spec** | **Test AUC** |
| --- | --- | --- | --- | --- | --- | --- | --- |
| S0 (Base) | Three-rater majority consensus (all 1,018 images) | 778 | 0.903 ± 0.013 | 0.880 ± 0.017 | 0.922 ± 0.010 | 0.852 ± 0.044 | 0.887 ± 0.022 |
| S1 (Strict majority) | Majority consensus, fully discordant cases excluded (n=1,016) | 776 | 0.903 ± 0.018 | 0.878 ± 0.024 | 0.923 ± 0.025 | 0.846 ± 0.075 | 0.885 ± 0.032 |
| S2 (High-confidence) | Three-rater complete agreement only (n=480) | 240 | 0.914 ± 0.019 | 0.889 ± 0.022 | 0.955 ± 0.023 | 0.803 ± 0.025 | 0.879 ± 0.018 |

All scenarios evaluated on the identical held-out test set (n=240, three-rater complete agreement).
Values represent mean ± SD across a single five-fold cross-validation run (5 folds), conducted specifically for the adjudication sensitivity analysis. These values therefore differ slightly from the primary model results in Table 1, which were derived from 10 repeated five-fold cross-validation (50 runs total).
A faculty-weighted adjudication scenario was not separately evaluated, as fully discordant cases comprised only 2 of 1,018 images (0.2%); the agreement stratum distribution is provided in Supplementary Table S1.

**Supplementary Table S3.** Comparison of different architectures for bowel-preparation classification (five-fold cross-validation, mean ± SD)

| **Model** | **AUROC** | **F1 score** | **Sensitivity** | **Specificity** | **Accuracy** | **Parameters (M)** |
| --- | --- | --- | --- | --- | --- | --- |
| Resnet-18 | 0.820 **±** 0.031 | 0.819 **±** 0.031 | 0.845 **±** 0.071 | 0.795 **±** 0.085 | 0.867 **±** 0.021 | 11.2 |
| Resnet-34 | 0.815 **±** 0.036 | 0.814 **±** 0.036 | 0.841 **±** 0.069 | 0.789 **±** 0.080 | 0.866 **±** 0.024 | 21.4 |
| ResNet-50 | 0.807 **±** 0.042 | 0.805 **±** 0.044 | 0.841 **±** 0.062 | 0.773 **±** 0.108 | 0.861 **±** 0.018 | 23.8 |
| DenseNet-121 | 0.831 **±** 0.032 | 0.830 **±** 0.031 | 0.856 **±** 0.055 | 0.806 **±** 0.082 | 0.875 **±** 0.021 | 7.1 |
| Densenet-161 | 0.823 **±** 0.031 | 0.823 **±** 0.029 | 0.860 **±** 0.052 | 0.787 **±** 0.085 | 0.875 **±** 0.022 | 26.8 |
| Densenet-201 | 0.831 **±** 0.032 | 0.830 **±** 0.031 | 0.856 **±** 0.055 | 0.806 **±** 0.082 | 0.875 **±** 0.021 | 18.3 |
| **DenseNet-201 + FPN (Proposed)** | **0.886 ± 0.017** | **0.882 ± 0.021** | **0.931 ± 0.025** | **0.801 ± 0.034** | **0.865 ± 0.019** | 46.4 |

Comparison of classification performance across convolutional network backbones.
Values represent mean ± standard deviation across a single five-fold cross-validation run, conducted for architecture selection purposes. These values differ from those reported in Table 1 and Supplementary Table S7, which were derived from 10 repeated five-fold cross-validation (50 runs total) for final model evaluation.
The proposed **DenseNet-201 + FPN** model achieved the highest AUROC and balanced precision–recall trade-off, demonstrating the advantage of multi-scale feature aggregation for real-world patient-generated images.

**Supplementary Table S4.** Distribution of samples across the held-out test set and the five stratified cross-validation folds.

| **Split** | **Total n** | **Not clean** | **Clean** |
| --- | --- | --- | --- |
| Held-out test | 240 | 175 | 65 |
| Fold 1 validation | 156 | 91 | 65 |
| Fold 2 validation | 156 | 91 | 65 |
| Fold 3 validation | 156 | 91 | 65 |
| Fold 4 validation | 155 | 91 | 64 |
| Fold 5 validation | 155 | 90 | 65 |

**Supplementary Table S5.** Estimated PPV and NPV of the DenseNet-201 + FPN model across a range of not-clean prevalence values.

| **Prevalence of not-clean (%)** | **PPV** | **NPV** |
| --- | --- | --- |
| 10% | 0.409 | 0.990 |
| 20% | 0.609 | 0.978 |
| 30% | 0.728 | 0.962 |
| 40% | 0.806 | 0.942 |
| 50% | 0.862 | 0.916 |
| 60% | 0.903 | 0.879 |
| 70% | 0.936 | 0.824 |

PPV and NPV were estimated using fixed sensitivity (0.922) and specificity (0.852) derived from the mean performance across five-fold evaluation on the held-out test set, applied across a range of hypothetical prevalence values using Bayes' theorem. At the observed prevalence in this dataset (61.8%), estimated PPV and NPV were 0.910 and 0.871, respectively.

**Supplementary Table S6.** Per-fold confusion matrix and derived metrics of the DenseNet-201 + FPN model on the held-out test set (n = 240).

| **Fold** | **TP** | **FN** | **FP** | **TN** | **Sensitivity** | **Specificity** | **PPV** | **NPV** | **Accuracy** |
| --- | --- | --- | --- | --- | --- | --- | --- | --- | --- |
| Fold 0 | 163 | 12 | 9 | 56 | 0.931 | 0.862 | 0.948 | 0.824 | 0.913 |
| Fold 1 | 159 | 16 | 9 | 56 | 0.909 | 0.862 | 0.946 | 0.778 | 0.896 |
| Fold 2 | 163 | 12 | 14 | 51 | 0.931 | 0.785 | 0.921 | 0.810 | 0.892 |
| Fold 3 | 162 | 13 | 6 | 59 | 0.926 | 0.908 | 0.964 | 0.819 | 0.921 |
| Fold 4 | 160 | 15 | 10 | 55 | 0.914 | 0.846 | 0.941 | 0.786 | 0.896 |
| Mean ± SD | - | - | - | - | 0.922 ± 0.010 | 0.853 ± 0.044 | 0.944 ± 0.013 | 0.803 ± 0.021 | 0.904 ± 0.012 |

TP = true positive; FN = false negative; FP = false positive; TN = true negative; PPV = positive predictive value; NPV = negative predictive value.
Results are from inference on the held-out test set (n = 240, drawn from three-rater complete-agreement cases) using each fold's best-epoch checkpoint from the stratified five-fold cross-validation (seed = 1004). All five models were applied to the identical held-out test set; the mean ± SD represents the average of these five per-fold predictions, not an average over different test splits.
Positive class = not clean (n = 175); negative class = clean (n = 65). The wide SD for specificity (±0.044) relative to sensitivity (±0.010) reflects the small size of the clean class (n = 65), where each misclassified image shifts specificity by approximately 0.015 (1/65).

**Supplementary Table S7.** Ablation study of the effects of random oversampling, smoothing weight decay, and feature pyramid network (FPN) components on bowel-preparation classification (five-fold cross-validation, mean ± SD).

| **Random oversampling** | **Smoothing weight decay** | **FPN** | **AUROC ± SD** | **F1 Score ± SD** | **Sensitivity ± SD** | **Specificity ± SD** | **Accuracy ± SD** | **Comment** |
| --- | --- | --- | --- | --- | --- | --- | --- | --- |
| ✗ | ✗ | ✗ | 0.880 ± 0.058 | **0.861** ± 0.0**67** | **0.916** ± 0.0**30** | **0.845 ±** **0.058** | 0.897 ± 0.021 | Baseline DenseNet-201 only |
| ✓ | ✗ | ✗ | 0.874 ± 0.024 | 0.867 ± 0.024 | 0.915 ± 0.031 | 0.834 ± 0.055 | 0.893 ± 0.021 | +Class imbalance correction |
| ✓ | ✓ | ✗ | 0.883 ± 0.023 | 0.877 ± 0.023 | 0.923 ± 0.032 | 0.843 ± 0.053 | 0.902 ± 0.020 | +Regularization effect |
| ✓ | ✓ | ✓ | **0.886 ± 0.031** | **0.882 ± 0.026** | **0.931 ±** **0.031** | 0.840 ± 0.072 | **0.906 ± 0.020** | **Full configuration (final model)** |

Values represent mean ± standard deviation (SD) from 10 repeated five-fold cross-validation experiments (50 runs total) on the development set (n = 778), consistent with the primary model evaluation reported in Table 1.
Stepwise inclusion of random oversampling, smoothing weight decay, and FPN led to progressive improvements in classification metrics, with the full configuration achieving the highest AUROC and balanced precision–recall trade-off, designated as the primary model for subsequent analyses.

**Supplementary Figure S2.** Calibration plot of the DenseNet-201 + FPN model on the held-out test set (n = 240).


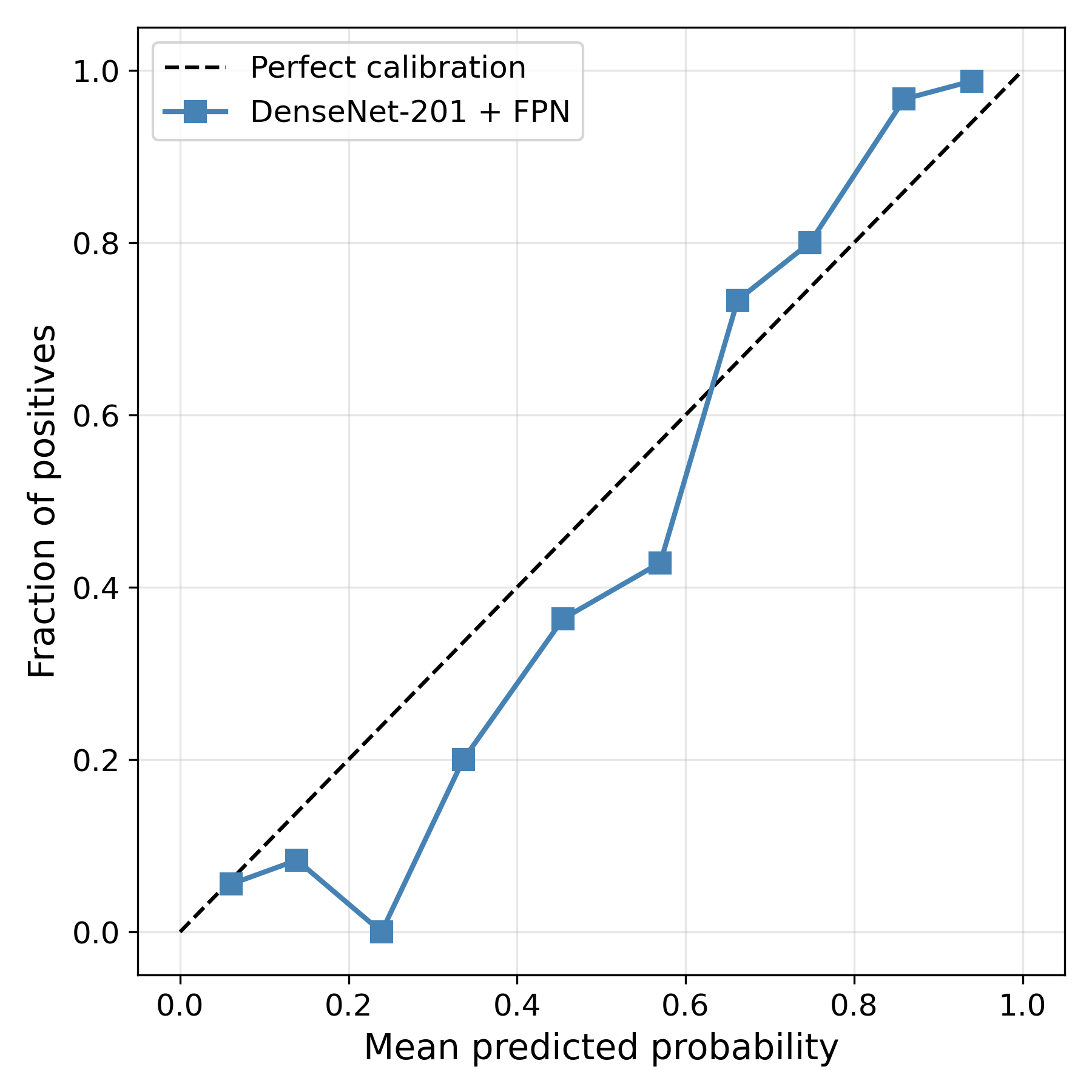


The dashed line represents perfect calibration (predicted probability = observed frequency). The solid line shows the model's calibration curve across ten equal-width probability bins. The overall Brier score was 0.077, and the calibration intercept of −0.099 indicated minimal overall probability bias. The calibration slope of 1.566 suggests moderate overconfidence, whereby predicted probabilities were more extreme than observed outcome frequencies. Minor deviations in the low-probability region reflect sparse bin occupancy. As this model operates as a threshold-based binary classifier, the observed overconfidence is unlikely to materially affect its intended triage output. Post-hoc recalibration (e.g., Platt scaling, temperature scaling) was not applied; its implications for probability-dependent clinical decisions are discussed in the Limitations section.

**Supplementary Figure S3.** Representative Grad-CAM examples showing successful and failed localizations

| (a) | 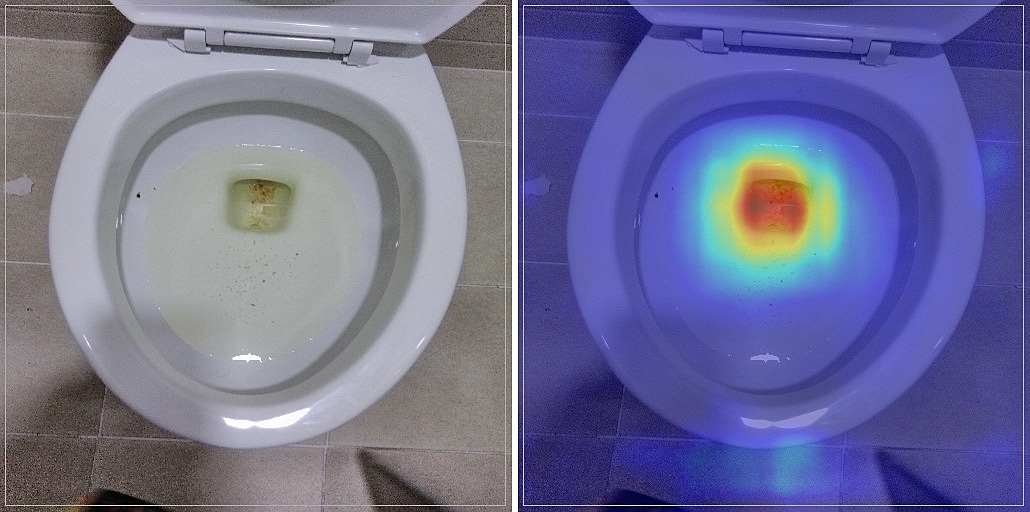 |
| --- | --- |
| (b) | 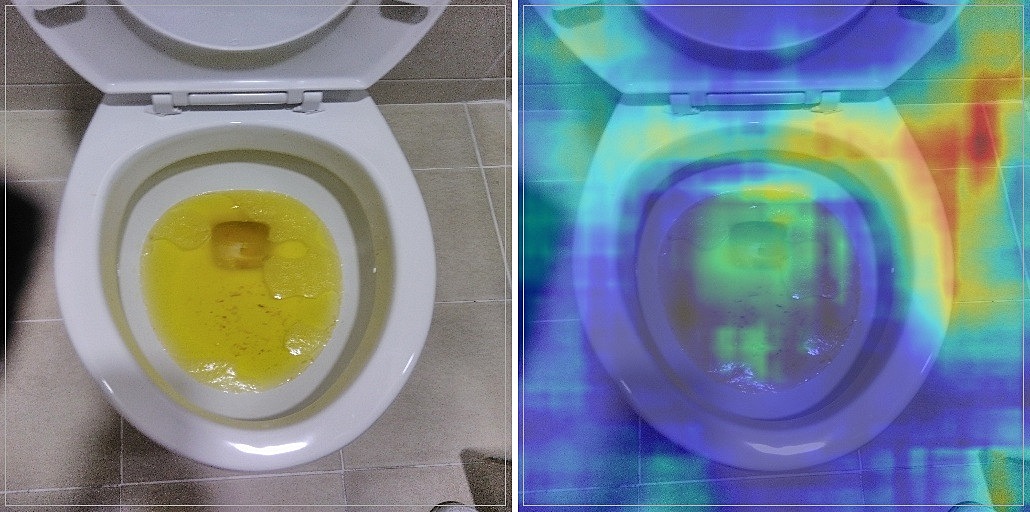 |

Comparison of successful and failed Grad-CAM visualizations for bowel-preparation image classification.
(a) shows a successful case in which the model’s attention correctly focused on clinically relevant regions, including residual stool and water turbidity inside the bowl.
(b) illustrates a failure case where activation maps were diffuse and mis-localized over the background or toilet rim areas, failing to capture the true regions of interest.

A clear contrast is observed between the two cases: successful maps align closely with clinician reasoning, whereas failed maps reflect sensitivity to lighting variation and frame occlusion. These findings indicate that the proposed model generally demonstrates strong explainability, but continuous visual auditing is needed to ensure robust interpretation under diverse conditions.

**Supplementary Figure S4.** Representative false positive and false negative cases from the test set.

| (a) | **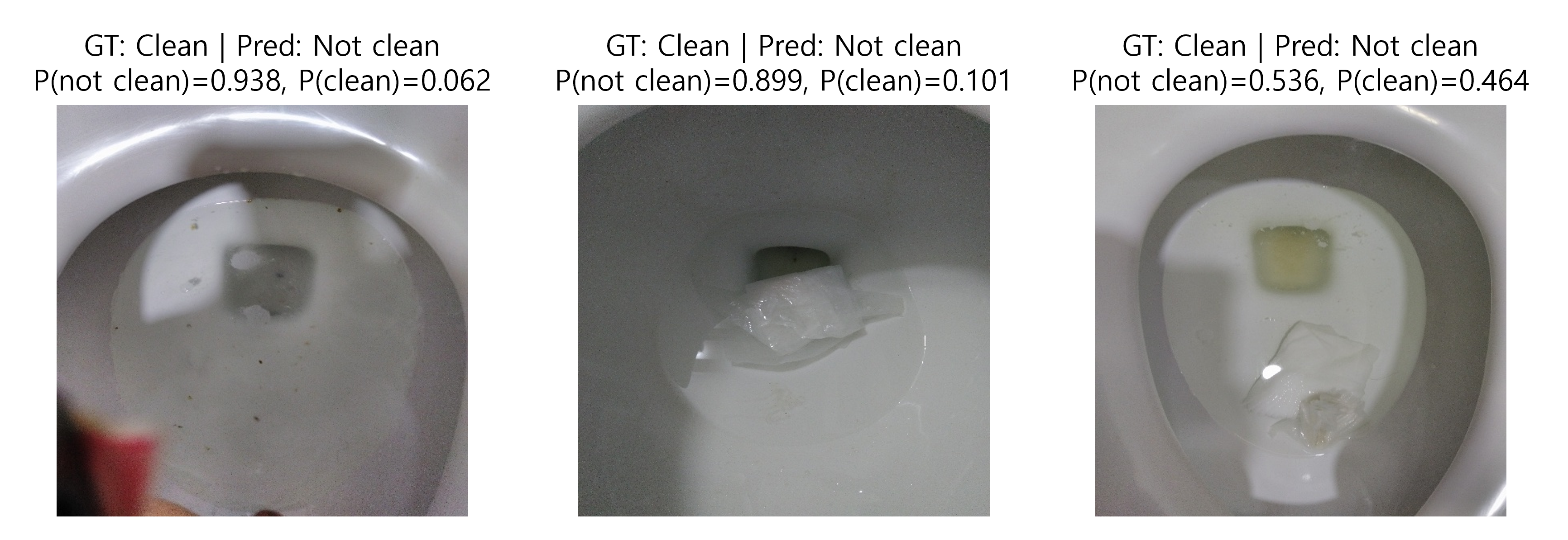** |
| --- | --- |
| (b) | 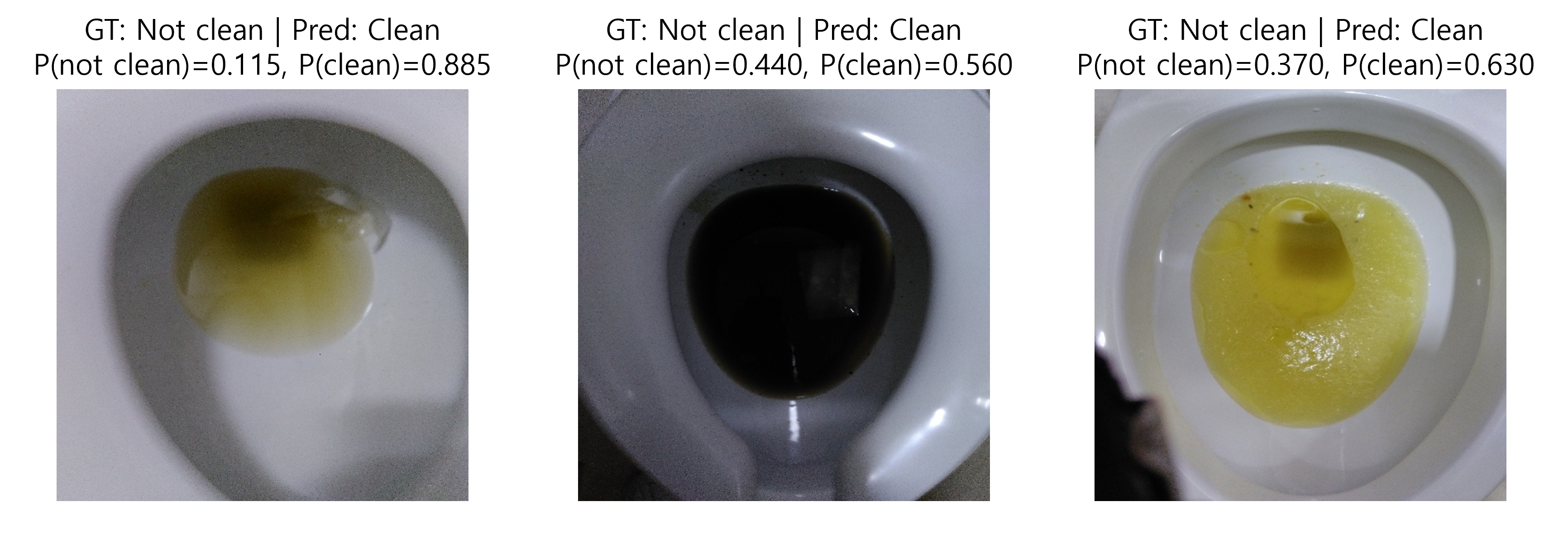 |

GT, ground truth; FP, false positive; FN, false negative; P(not clean), predicted probability of inadequate preparation; P(clean), predicted probability of adequate preparation.
(a) shows representative false positive (FP) cases in which the model predicted inadequate preparation despite adjudicated adequate status. Interfering elements such as foam or tissue paper, combined with discoloured and turbid water, likely caused the model to misclassify these visually complex but clinically acceptable cases.
(b) illustrates representative false negative (FN) cases in which the model failed to detect water turbidity and residual soiling present in the images, leading to erroneous clean predictions.

Taken together, these errors suggest that the model is susceptible to visual distractors in borderline-clean cases and to underdetection of turbidity when residual soiling is present. Predicted probabilities are re-normalised to sum to 1.0 across the two classes.

**Supplementary Figure S5. Threshold analysis of the DenseNet-201 + FPN model on the held-out test set.**


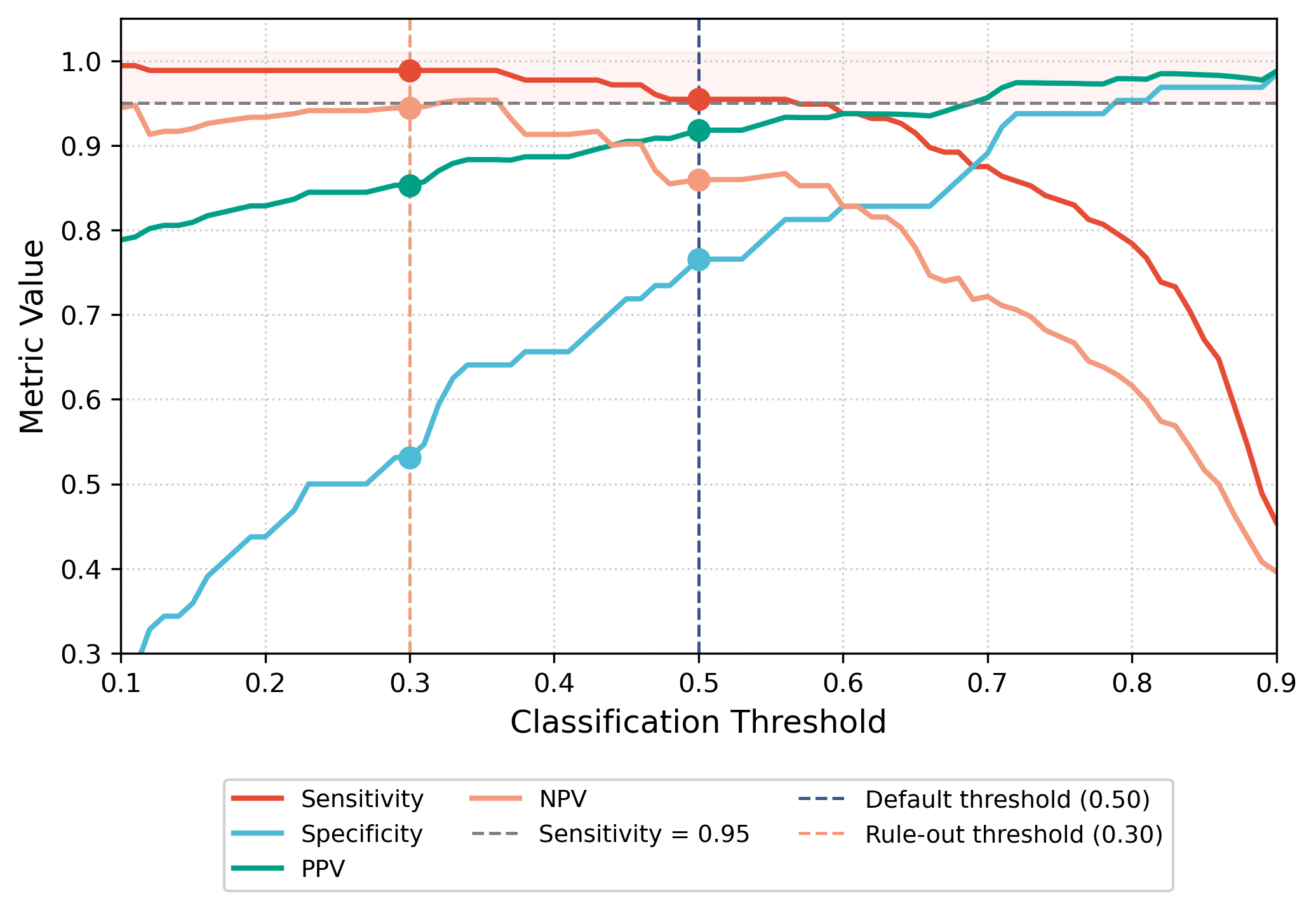


Sensitivity, specificity, PPV, and NPV are shown across classification thresholds ranging from 0.10 to 0.90 (n = 240).

The horizontal dashed line indicates the sensitivity = 0.95 criterion. Vertical dashed lines indicate the default threshold (0.50) and the recommended low-threshold operating point (0.30), which achieves sensitivity ≥ 0.95 while maximizing specificity. The shaded region denotes the sensitivity ≥ 0.95 zone.
